# Supplementary material for: Choice of method of place cell classification determines the population of cells identified
Source: PLoS Comput Biol. 2021 Jul 8;17(7):e1008835. doi: 10.1371/journal.pcbi.1008835 (PMC8291744; doi:10.1371/journal.pcbi.1008835)
Supplement: Attachment — Submitted filename: response_to_reviewers.docx [file pcbi.1008835.s010.docx]

Response to Reviewers

We would like to thank the reviewers for their time and comments on our manuscript and address these all individually below. The process of addressing these comments has improved the scope and relevance of our paper, and we are very grateful for the constructive input. We respond to the individual points in detail below, but first want to highlight the main improvements we have implemented. First, we agreed with the comment that a fixed velocity threshold would be preferably, so have recalculated all the results using a 2 cm/s threshold, changing all the figures as appropriate. We have also added in an additional method, again as result of an excellent suggestion from one of the reviewers, which has been incorporated into all figures and discussion as appropriate. In addition, we performed a new simulation where we randomly varied several model place cell properties simultaneously, resulting in a new main figure (Fig 4), and also made two new supplementary figures (S3 and S4 Figs) to provide more detail about the properties of the simulated cells, and allow experimenters to better compare their real data to our simulations.

Our specific responses to individual comments are below.

Reviewer #1: The paper discusses methodological considerations in classifying hippocampal place-cells in two-photon calcium imaging data. The authors present a thorough analysis and discussion of 3 methods and their comparison on a modeled dataset, manipulating for several parameters that may arise in real data. Lastly, the authors compare the methods when applied on real data. They find striking differences between the methods and differing detection characteristics/overlap for place cells, highlighting the need for standardization. The article is well written and presented with only a number of minor issues affecting readability and clarity.

We thank the reviewer for their positive assessment of our paper.

Comments:

In the section discussing differences between simulated vs. real data results, the authors propose that the difference in reported place-cell numbers in the stability method may be due to changing place-field characteristics over the time course of a recording. However, in simulated results from Fig 3, it was reported that stability would still report equal or higher sensitivity when variance increased or when reliability decreased. After referring to Fig. S6, the fluorescence changes that may factor into decreasing number of detected place-cells using the stability method in the real data appear to be similar to the simulated manipulations in Fig 3, yet the findings disagree. Clarification is needed.

The reviewer is correct. The Stability method does indeed seem to be less sensitive to changes in reliability and variability as we measured it in our modelled datasets. However, as the examples in S Fig 8 demonstrate, the Stability method seems to particularly miss examples where the variability and/or reliability is structured, such as the cells having a place field only in the first or second half of the session, or multiple place fields existing concurrently. As the reliability and variability was varied randomly in the simulations, they did not capture this structured variation. We have now clarified this in the text:

“This type of structured variation in the place field location was not captured by our simulations of place field firing, where reliability and variability were varied randomly, which did not affect the ability of the Stability method to detect place cells.”

The authors should provide further details on their linear mixed-effects modeling, including package, package version, and variables.

We have added the effects included in the model in the text, and have included the package and package version in the methods section:

“Linear mixed models were performed in R using the lme4 package (version 1.1-23).“

Fig 3, panels B, C, E, F missing legend

We have added the missing legend in the new version of the figure.

Fig 4, panel C. The figure indicates that this includes all cells in the recording. However, there seems to be a consistent fluorescence of location at (almost) each cell. Are these only the cells identified by the one (or all) of the detection methods? The authors should clarify.

Fig 4 (now Fig 5) shows the normalised fluorescence for all cells, including those that were not classified as place cells by any method, ordered by the location of the highest fluorescence. This ordering makes the cells seem more place cell-like than they are, and closer inspection of individual cells will reveal that many are indeed not place cell-like (e.g. cell 1, 13, 14, to point out a few). To clarify this we have added the word “all” to the Fig 4 legend as follows:

“*(C) Fluorescence maps over location of* ***all*** *ROIs in an example recording.”*

*We have also added further clarification in the text:*

*[line 378] “The properties of these cells can be seen in an example recording showing the fluorescence maps of all cells, ordered by location (Fig 4C). In addition, the populations of cells identified by each of the methods are shown (Fig 4D-G). Although no ground-truth exists for what should be included as a place cell, most identified cells mostly seem to have clear place fields, suggesting classifications are largely appropriate.”*

Fig4, panel G, the image shows more areas detected as place-cells with the stability method, which doesn't agree with earlier panels. Why is that the case?

This particular dataset was selected as it had the most Combination place cells, but also happened to have an increased number of Stability place cells, and was therefore not typical compared to the other datasets. After rerunning the methods with the updated velocity threshold, the same dataset now more closely reflects the typical number of place cells selected by each of the methods.

Fig S1 is quite informative but would benefit from slight clarification linking the text-boxes to exact subplots.

We have now added references to the subplots in the text boxes.

Fig S3, left panel is missing the peak method line. If overlapping, it should be adjusted or noted in caption.

Thank you for the comment. We have now noted in the relevant figure legends where the Peak method line (and indeed the new Information method line) are overlapping (Fig 2, S5 Fig, S7 Fig).

The paper discusses differences in methodology and their critical effects on results and potential inconsistencies in replication. I agree with the authors and would strongly recommend sharing the code for the 3 compared methods. Having access to the the code will ensure that any implementation idiosyncrasies are accounted for and will be a great asset for future work and replication to supplement the model-data generation scripts they have provided.

Thank you for this recommendation, we have now included code to run all four methods in our GitHub repository (https://github.com/DoriMG/place_cell_methods) in addition to the model data generation.

Reviewer #2: The authors presented a robust comparison between three different methods for classifying place cells based on their calcium activities. After establishing the impact of several parameters on the performance of the three approaches in an in-silico environment, they used them to classify place cells from the CA1 distal portion of the hippocampus of mice stimulated in a 1D VR environment. They found that the three methods identified nearly independent population of place cells in vivo, raising concerns about what we should use to classify and how we should define place cells. Overall, the manuscript is linear, clear and relevant. I have only minor comments and clarifications.

We thank the reviewer for their helpful comments and positive appraisal of our work.

• Could you please provide the codes of the different methods? In alternative, pseudocodes, in addition to Suppl. Fig.1 (which is very clear), could help the reader to easily reproduce your approaches.

Thank you for this recommendation, we have included code to run all four methods in our GitHub repository (https://github.com/DoriMG/place_cell_methods) in addition to the model-data generation.

• It is not clear to me how much of the “excess activity variance” behavior (place cells also fire outside the place field) was modeled in the in-silico dataset.

In our initial simulations, we only added Poisson noise, which provided low amplitude noise, but no calcium peaks outside of the place field. We did perform a simulation where we also added model calcium peaks to the activity of the non-place cells (S7 Fig D-F), and we have expanded on this now through an additional simulation where out of field calcium peaks were also added to the model place cells (S7 Fig J-L). This led to an increase in sensitivity for the Combination method, especially at higher noise levels, and increased specificity for the Stability method.

We have clarified this further in the text:

[line 164]: “Poisson noise, which in the default model had an average fluorescence 0.18% of the default peak height, was then added to the traces all cells.”

[line 421]: “Lastly, adding in random calcium peaks to the place cells in addition to the non-place cells caused a similar increase in specificity for the Stability as adding them only to non-place cells (S7 Fig J-L).”

We have also further discussed these results in the supplementary information (S7 Fig).

• Considering that a place cell fires about 50% of the time spent in the field place, how this variability was introduced in the in-silico dataset? If it was not included explicitly in the model, do you think that it could affect the performance of the Peak method in particular?

This is an interesting point. We did not explicitly model the case where, at any point in a place field, there is a particular probability of firing, but the impact this would have on firing is reflected in the variables we did consider, namely the reliability and variability manipulations. The dataset with 50% reliability (Fig 3B-C) reflected cells only being active in 50% of the traversals (even though they do traverse the place field in each traversal). To some extent this is also reflected in the variability, in which the position of the place field is varied. Thus, firing in a variable place field location produces a similar effect to a cell only firing a proportion of the time it spends in a stable place field. The combination of these two factors (as shown in Fig 3G and H) shows that the Peak method still has a high sensitivity even at a reliability and variability of 50%.

• You modeled the place cells as independent from each other and from the non-place cells. Considering that the network state is predictive of the place field response of a place cell, how much do you think that the performances of each method were biased by the absence of a network?

Again, this is an interesting question. In the case of the Peak, Combination and Information methods, any network effects are unlikely to bias the classification of the cells, as each cell is merely compared to shuffled versions of itself. Hence, other dynamics present across the population will not affect how each individual cell is classified. However, for the Stability method this could affect the classification, because in this method each cell is compared to other cells, with the assumption that a cell’s place field in the first half is more likely to correlate with its own place field in the second half of the session, than that of any other cell. However, if the cells are more correlated due to network dynamics, the intracell correlation will be lower relative to the intercell correlation, so fewer cells overall will be classified as place cells, resulting in a lower sensitivity. Conversely, as the intercell correlation is higher, fewer non-place cells will also be considered place cells, resulting in an increased specificity. To consider this point, we have added the following text to the discussion:

[Line 674]: “Lastly, in our simulations we modelled each cell independently. By modelling our cells this way, we are potentially missing out on network dynamics within the population, where cells fire dependently of each other, or are synchronously affected by a third factor, such as global theta oscillations. However, because each cell is individually evaluated in the Peak, Combination and Information methods, these methods will not be affected by such dynamics, and as such adding network dynamics to our models would not affect the results. In the case of the Stability method, the cells are compared to shuffles across the population, and both the sensitivity and the specificity of this method will very likely be affected. An increased correlation across the population will cause an increase in the shuffled correlation, thus a higher threshold overall for all cells. If this increase in threshold is large enough, this will reduce the sensitivity of the method, classifying fewer place cells, while increasing the specificity, meaning fewer non-place cells will be falsely marked as place cells. Indeed, we saw a similar effect on specificity for the Stability method, when we all place fields in the population were in the same location, thus causing an increased correlation across the population (S7 Fig I). In addition to correlations of activity across the population of pyramidal cells, the activity of a cell in a given traversal could depend on its activity in the previous traversal (e.g. if a cell fires in traversal n, it might be more likely to fire in traversal n+1). This will only affect the Peak, Combination and Information methods in the same way as they are affected by the reliability, as they classify place cells based on the average fluorescence map across all traversals, and do not take individual traversals into account. However, the Stability method may be affected if such dependency between traversals causes a cell to lose or gain a place field, or if the place field shifts position between the first and second half of the session, as demonstrated in S8 Fig. Therefore, if one expects strong network dynamics to be present in a population of place cells, we recommend using caution when employing the Stability method.”

• In consideration of the role of theta rhythms on the activity of the place cells, in which measure do you think that the presence of such signature could affect the performance of the methods?

The theta rhythm operates at around 4-7 Hz. Since we image at 7.51 Hz, and use a calcium indicator with a decay rate of about 0.7s, it is very unlikely that we will see any effects of theta rhythms. As such, the activity of place cells relative to the theta rhythm, in the form of phase precession or phase coding, will not be observable in our data, and activity from the various phases of the theta cycle will be averaged out in the calcium signal. As such, we do not believe phase precession or phase coding will impact the performance of our methods.

For theta rhythms possibly coordinating firing across the network, the same principles as for the previous comment apply, namely that the Peak, Combination and Information methods will not be affected by this, while the Stability method possibly will have a lower sensitivity. The text added now addresses this potential effect of theta.

• Considering the two previous comments, could you comment about your decision to omit network behaviors in the model? I understand that the simulations must be simple and network effects are difficult to model, yet it would be important underline this lack in the manuscript in my opinion.

Given that the Stability method already does not perform well on our other manipulations, and the other three methods are unlikely to be biased by such network states, we had elected to keep our simulations as simple as possible and not include network states in them. However, on the advice of the reviewer, we have now added a small model where we tested the effect of all the place cells having the same place field location (S5 Fig G-I), to simulate the effect of increased correlation of firing across the population of cells. As expected, this did not have any effect on the Peak, Combination or Information methods. However, for the Stability method, this caused an increase in specificity, i.e. it classified fewer non-place cells as place cells. This effect occurs because the increased intercell correlation creates a higher overall threshold for cells to be included as place cells. Overall, it suggests that network dynamics could increase the performance of the Stability method, but would not affect any of the other methods. We have expanded on these considerations with the addition text added to the conclusion, as quoted above, as well as in the text:

[line 421]: “Lastly, adding in random calcium peaks to the place cells in addition to the non-place cells caused a similar increase in specificity for the Stability as adding them only to non-place cells (S7 Fig J-L).”

We have also further discussed these results in the supplementary information (S7 Fig).

• It is unclear to me if, in the Peak method, the rate map across locations is also calculated across transversal; since in Fig.3B the sensitivity of the Peak method showed a dependence to the % of reliability, I assume that the transversal are somehow considered. Could you please clarify it?

The fluorescence map (equivalent to the rate map) is calculated from average activity in each location, and traversals are not explicitly considered in this. However, with a lower reliability, the average activity will drop, as the cell is less likely to be active in the location (its place field) in which it is otherwise most active. This will also result in a lower peak relative to the shuffle (which will be dominated by background noise), resulting in a failure to be marked as a place cell. However, this only happens in very few cases when reliability is higher than 10%. We have now clarified this in the text:

[line 286]: “The effect of reliability on the Peak and Information methods comes not from any explicit comparison of the methods between traversals, but because unreliability decreases the size of the place field peak in the average fluorescence map, due to the existence of traversals in which the cell is inactive .”

• It is not clear to me how in the Stability method non-place cells can be detected (specificity about 80%) in the in-silico models if they are not firing. Do you have any explanation or am I missing something?

The non-place cells have background Poisson activity, but when averaged, the activity from the first and second half can still highly correlate (i.e. they are both flat lines). This correlation is compared to the intercell correlations across the overall population. Even if the correlation of the non-place cell is not as high as the place cells, it may still pass the threshold compared to the shuffled controls, thus leading to it falsely being classified as a place cell. This seems to happen in roughly 20% of the cases, hence the 80% specificity. In fact, when we add in additional calcium peaks outside of this Poisson noise, which decreases the amount of correlation between the two halves of the fluorescence maps for the non-place cells (S7D-F Fig), the sensitivity increases to levels similar to the other three methods.

We have clarified this in the text:

[line 406]: “Increasing the percentage of place cells and adding random calcium peaks to the non-place cells resulted in an increase in specificity (S7 Fig A-F), likely because it decreased the correlation of the fluorescence maps of the first and second halves of the sessions for the non-place cells.”

• In the in-silico dataset, the authors modeled 100 cells, which is reasonable and in line with the average number of neurons detected in vivo using optical approaches. In which measure the Stability method is affected by the number of total cells (since random cells are selected in the method)? Could this partially explain some decays in the performance of the approach? Could you comment?

We are sorry but we don’t quite follow the question. If the reviewer is asking whether looking at different numbers of cells affects the performance of the Stability method, we think this is unlikely, as both the number of place cells and the sensitivity and specificity values are relative to the total number of cells. In other words, increasing the number of cells (without adjusting the percentage of place cells), will likely cause an increase in the number of non-place cells being classified as place cells, but this number will stay proportionate to the total number of cells, thus the specificity will remain the same. However, altering the percentage of place cells (S7 Fig A-C) does cause an increase in specificity for the Stability method. This is likely because an increase in place cells cause an increased overlap in their place fields, and thus a higher intercell correlation. This will increase the average correlation of the shuffled control, thus increasing the thresholds for cells to be classified as place cells, ultimately decreasing the number of false negatives.

• For a given simulation, was each parameter the same across the place cells or it ranged (i.e. width=50 ± xx cm)? In other words, has the same Gaussian field been used for all 20 cells for a given simulation? If so, how much do you think that this homogeneity could bias the performances of the methods?

Yes, for a given simulation the same Gaussian field was used across all 20 cells, though the location of that field was spread out across the full environment. However, we have now added an additional figure (Fig 4), where we simulated populations of place cells where the parameters were not homogeneous across cells. Because we vary all parameters, rather than one at a time, in this simulation, we cannot precisely distinguish between the effect of homogeneity and the effect of the interaction of the parameters in this simulation. However, the results suggest that indeed the homogeneity affects the performance of the different methods. Most notably, the Stability method shows a lower percentage of cells classified as place cells, compared to the Peak and Information methods, suggesting an increased specificity compared to previous simulations, as well as a decreased sensitivity. We discuss these results in a new results section starting from line 314.

• Could you clarify in the manuscript the reasons why you used the 1D Gaussian model of the fluorescence of a place field? How much do you think that the use of a Gaussian bias the Peak method in particular?

We use a 1D Gaussian model largely as an approximation for the place field shape, as it allows us to parameterize our place fields into the peak, width and centre of each place fields, which are then easily manipulable. We believe the 1D Gaussian approximation is not dissimilar to the actual shape of place fields, although it ignores the presence of a skew, which is especially likely in experienced animals [1]. Introducing such a skew would likely minimally influence the Peak method, as these place fields still have a clear peak that will disappear in the shuffled version of the cell, thus still resulting in a high percentile score.

• Is the activity of the nth transversal modeled independently from the history of the activity of the nth-1 transversal? Do you think that activity-dependent effects could impact the performance of each method?

Yes, each traversal is modelled completely independently. This is mainly relevant for manipulations of the reliability and the variability. In case of the reliability, the traversals where a cell fires is selected based on the set reliability (%), rather than each traversal having a probability of being active. If there were activity-dependent effects (i.e. if a cell is more likely to be active in the n+1th traversal, if it was active in the nth traversal), we believe this would minimally impact the Peak, Combination and Information methods, as they only include a measure of the overall reliability (i.e. numbers of traversals active), but do not require sustained activity across subsequent traversals. However, it is more likely to affect the Stability method, as having such a dependence between traversals, is more likely to cause a difference between the first and second half of traversals, for example place cells that either lose or gain a place field throughout a session. We have shown that in the real data, these cells sometimes wrongly get classified as non-place cells by the Stability method (S6 Fig), so we would expect introducing similar properties to our simulated data would similarly reduce the sensitivity of the Stability method. In addition, if the dependence extends to place field location (for example the location drifts as a session progresses), the Stability method will also be affected, as this causes a decorrelation of the fluorescence maps of the first and second half of the session. To consider this point, we have added the following text to the discussion:

“In addition, the activity of a cell in a given traversal could depend on the activity in the previous traversal (e.g. if a cell fires in traversal n, it might be more likely to fire in traversal n+1). This will only affect the Peak, Combination and Information methods in the same way they are affected by the reliability, as they classify place cells based on the average fluorescence map, and do not take individual traversals into account. However, the Stability method may be affected if such dependency between traversals causes a cell to lose or gain a place field, or if the place field shifts position between the first and second half, as demonstrated in S8 Fig.”

• The authors distributed the place field centers evenly across the map; do you think that this could have an effect on the performance of the Stability method since each cell would have a significant overlap (higher correlation than random non-place cells or farther place cell in the field) of their place fields with their neighbors (Step 3 of the method)?

As the Stability method compares the correlation between the fluorescence map over the first and the second halves of the session within a cell, to the correlation between the first halves of all cells and the second half of randomly selected cells in the overall population, having the cell be active in the same (or similar) location(s) will increase the likelihood of getting a high correlation in the shuffle. This might be expected to cause false negatives, where a place cell is deemed not a place cell, because of this high threshold correlation. We have now added in a test (S5 Fig G-I), where we modelled all the place fields of the place cells all in the same location. In fact, this did not affect the sensitivity (i.e. the increase in correlation between shuffled controls – as place cells now correlate highly with other place cells – was insufficient to increase the number of false negatives). However, specificity increased, indicating that the overall increase in correlations in the shuffled controls did prevent more non-place cells from being classified as place cells. Thus an overlap of place fields does not negatively affect performance of the Stability method.

However, evenly distributing the place cells across the map most closely resembles previously studies, which show coverage of the environments and overlap between place fields. We have clarified this in the manuscript:

“For the model place cells each place field was modelled as a 1D Gaussian field centred at equidistant locations covering the entire environment, mimicking previously reported place cell populations [10,36] (Fig 2A)”

• Was the noise added in the model able to influence the Gaussian field distribution and, more broadly, the probability of a cell to fire or not?

In the default condition, the noise was much lower than the Gaussian field peak, so it was not able to influence this distribution. We have now clarified this further in the text:

[line 164]: “Poisson noise, which in the default model had an average fluorescence 0.18% of the default peak height, was then added to the traces all cells.”

However, in the model where we vary the peak size, as shown in Fig 2, at lower peak sizes this amplitude of noise will influence the Gaussian field distribution, resulting in a decrease in sensitivity at very low peak sizes for all methods. We have now performed a further simulation, where we added in additional calcium peaks as noise in addition to the Poisson noise to the place cells (S5 Fig J-L). Adding in these out of field peaks causes an increase in specificity of the Stability method, and an increase in sensitivity of the Combination method, ultimately resulting in an indistinguishable performance between the four methods at a noise level of 0.01-0.0125 peaks per frame. This is notably higher noise than our measured average of 0.0062 peaks per frame in the real datasets, at which rate, the Combination method performs at a lower sensitivity than the other methods.

• Did you perform a set of simulations in which the Gaussian was different for each cell, thus varying in a narrow range width, peak, % reliability and % variability? Even though varying one parameter at time is instrumental in dissecting single contributions, do you think that it could be beneficial to model a more realistic scenario to test the performance of the different methods? You did already something very similar in Supplementary Figure 4 but varying only width and peak. It would be interesting to understand in this scenario the percentage of cells identified from each method and the percentage of cells identified from more than one method (and maybe help to clarify also the discrepancies highlighted in the real data).

This was a really interesting point to address! We have now added an additional figure (Fig 4), where we simulated populations of place cells where the parameters are varied per cell, as you suggested, and have added a section to the results explaining and discussing the results from this figure (from line 314). Overall, a similar trend in the percentage of cells emerges to the real data, but the overlap is much higher in this simulation compared to that in the real datasets. in addition, this new figure shows how the various parameters affect the percentage of cells classified as place cells when they interact within the population. In general the direction of the effect of random changes in a given parameter on a background of random changes in other place field parameters is as expected from the effects of systematically varying this parameter. The notable exception is an interaction which emerges between the effects of varying place field width and variability on detection by the Combination method. This results in the absence of a distinct range of place field widths which are detected by the Combination method, which instead now performs more like the other methods (but with a lower overall sensitivity; compare Figs 2C and 4E).

• In the results section the authors correctly pointed out that the spatial information carried by place cells detected with the Peak method can derive also from other sources, yet in the discussion the repeatedly used this argument in favor of the Peak method. It sounds odd to me (and I personally agree with the caution mentioned in the results section).

We are sorry that our position on this was unclear. Indeed, we are more in favour of the stance that equating place cells to high spatial information is not necessarily favourable, and we have now rephrased this in the conclusion, especially when contrasting the Peak method with the newly introduced Information method, which does make this assumption. We have now clarified this point at several points in the manuscript

[Line 696]: “Overall, we found that the Peak and Information methods both demonstrated a high selectivity and specificity for selecting model place cells that was robust to moderate changes in place field properties but decreased appropriately as the reliability and variability for the place field decreased. It detected more place cells in a real dataset and these cells carried more mutual information about location than non-place cells, unlike the other methods. Because of its simplicity and lack of assumptions about the spatial information held by place and non-place cells, for most experimental designs we would recommend use of the Peak method for classifying place cells in calcium imaging data.”

And [line 626]: “However, compared to the Information method, the Peak method does not inherently make assumptions regarding the spatial information held by potential place cells. In addition, the Peak method has also been successfully employed to detect place cells in electrophysiological recordings [24], making it widely applicable beyond two-photon recordings. Therefore, we believe the Peak method will usually be the preferred method.”

• In the in vivo experiments each mouse had 2 sessions; did a session-dependent effect emerge in the place cell classification?

This is an interesting question which we have now investigated by including the mouse ID as a random effect and the session number as a nested random effect within our linear mixed model to control for this. If we explicitly test for effects of session or mouse, we find that the session number itself had no effect - there was no difference between session 1 and 2 (p=0.4053), nor was there a significant difference between the individual mice (p=0.6459), so there was no alteration in place cell classification with multiple sessions per mouse. We have added text to describe the statistical testing using a linear mixed model as follows:

[line 380, 458, 600]: “linear mixed-effects model with method as a fixed-effect and mouse ID, with session number nested, as random effects”

• Personal note: for a given figure, in my opinion it would be clearer to have both the sensitivity and specificity graph y-axis ranging from 0 to 1. It could give the reader an immediate sense of the fact that usually specificity is more robust.

Thank you for this excellent suggestion, we have now changed the graph y-axis, as well as the colour maps, on all graphs to range from 0-1.

Reviewer #3: Summary:

Place cells are hippocampal pyramidal neurons whose activity is modulated by the position of an animal in its environment. Collectively, an ensemble of place cells can be used to decode the position of an animal in its environment. Recently new calcium imaging techniques have been used to study place cells. They notably allow direct cell visualization, facilitating detection across several days or even weeks and recording of a large neuronal population (up to a thousand cell). However, calcium indicators only indirectly report single cell activity and this could dampen proper analysis of place cells.

Several methods can be used to detect place fields ranging from simple ones based on a fixed threshold applied to activity averaged over several trials to more sophisticated ones based on comparisons with shuffled data. The present study aims at comparing different methods on a model dataset and real calcium imaging data. We believe this approach could be useful to decide which method should be used depending on the specific experimental design.

We thank the reviewer, we also hope it will prove useful to researchers in the field in choosing a method, as well as be informative as to the limitations of using any given method.

1. Authors should justify the three methods compared. They do not appear to be widely used in the field. One (The combination method) was used essentially by a single lab (Tank lab) another one (the stability one) was used only in an unpublished work (O’Leary et al., SFN abstract 2019) and the third one (the peak method) has been used only in one study. To be useful for the community I think this work should include more widely used methods for detecting place fields in calcium imaging data (e.g. from the Ziv or Lonsonczy labs).

The Combination was initially developed in the Tank lab, but has since been used in several papers [2,3], from a number of different labs. We therefore do think this is a very relevant method to be testing. The Stability method in its current form (i.e. on fluorescence data) has indeed only been used in unpublished work, but was based on a widely used method to characterize cells in electrophysiology (e.g. [4,5]). The Peak method was included largely for its simplicity, and the fact it was previously employed on electrophysiological data, making it widely applicable. We have now included a fourth method, the Information method, which is a slight adaptation of the method used by the Ziv and Schnitzer labs, as suggested by the reviewer. We show that this method performs almost identically to the Peak method, though overall selecting a higher number of cells as place cells, likely due to the lower threshold (95^th^ percentile, compared to 99^th^ percentile for the Peak method). However, this method does inherently assume place cells to contain more spatial information, while the Peak method makes no such assumption, which is why we still prefer the Peak method. We have amended all figures to include this new method and the text in multiple locations where appropriate, including the following, relating to our overall conclusions about the Information method:

[line 591]: “The increased selectivity and specificity of both the Peak and Information method when using model data, leads us to conclude that these methods will usually be the best for detecting place cells in calcium imaging data. However, compared to the Information method, the Peak method does not inherently make assumptions regarding the spatial information held by potential place cells.”

We have now also added further justification for the selection of these four methods in the text:

[line 135] “We selected the four methods for varying reasons. The Peak method was originally applied to electrophysiological data, and its simplicity allows for the application to fluorescence data with minimal adaptations. Similarly, the Stability method was based on methods used for characterising place cells in electrophysiological recordings (e.g. [3]), while the Information method, or variations thereof, has previously been used to detect place cells in both electrophysiological [26] and fluorescence data [27]. This makes these three methods promising candidates for application across the two recording paradigms. In contrast, the Combination methods was designed specifically for fluorescence data, and has been widely employed in the field (e.g. [11,20,25]).”

2. As discussed in the manuscript the ultimate question is what is a place cell (what are their core properties) with detection methods differing accordingly. The most prevalent property of place cells is that their activity should be modulated by the position of the animal in the environment in a consistent way. So, a bump of activity should clearly be observed specifically and consistently when animal is in a specific location in the track. In terms of consistency, the combination method whose sensitivity slowly builds up with increasing consistency looks more appropriate. Could the authors comment on that?

We agree with the reviewer that a specific and consistent increase in activity should ideally be observed at the same location. However, it should be noted that our modelled place cells are ‘perfect’ example place cells, and have very low noise compared to actual fluorescence recordings. Thus, for the Combination method to not detect all place cells, even at high reliability and low variability scores, speaks against this method. Indeed, when testing the method on the real datasets, the Combination method detects many fewer cells compared to the other methods, potentially missing cells that most researchers would deem place cells.

Overall, as we try to convey in our manuscript, the choice of classification method is eventually subject to personal preference, and one could definitely prefer a more conservative method, which only selects very consistent place cells, but potentially misses out other cells that could very well be considered to be place cells. We hope our manuscript helps in facilitating this decision in the future, and, by making explicit the different properties selected for by the different methods, provides a benchmark to then compare research using such a method with studies using other methods.

3. Along the same line place cells with a place field width as large as the all environment (Fig. 2) probably convey few information about animal location and it is questionable whether these cells can be called place cells. In that respect a method like the combination method that does not detect place cells with very large place fields is probably more relevant than methods that readily detect those place cells but this interpretation is not conveyed in the manuscript nor discussed.

As the widths are modelled by a Gaussian, even at the higher widths (up to 200 cm) in our model, the place fields still have a distinctly place cell-like shape. We have now added S4 Fig, with examples of place fields of varying widths, to illustrate this. We therefore do not think the detection of these cells is irrelevant to the study of place cells, and they should probably be included in the population of potentially classified place cells. As various paradigms, including the size of the environment and the location of the cell in the hippocampus, can cause a variety of place field sizes, we believe that restrictions should be used cautiously, as they may exclude interesting dynamics. Of course, when using the Combination method, one can employ thresholds appropriate to the paradigm. As discussed above, we hope that this manuscript will make explicit the impact of using a given method on the cells classified or not by that method. If people do not want to include cells such as that in S4 Fig D as a place cell, then this manuscript will help them understand what method best to use.

4. The drop of specificity of the stability method with the number of traversals is puzzling. From the example cells in Fig. 1E firing appears to be very stable between two halves of the recording for at least 50 trials. Why is this drop in specificity observed?

With an increase in traversals, the Poisson noise of the non-place cells will increasingly average out over the traversals, thus resulting in a flatter ‘place field’ (i.e. the same activity in every location). As this ‘place field’ becomes flatter, the first half and second half become increasingly correlated, resulting in non-place cells being wrongly classified as place cells, and thus a drop in specificity. This affects the Stability method in particular, as it has no requirement for the potential place cells to show peak-like activity, as long as the activity is correlated between the first and second half of the session.

We have now clarified this in the manuscript:

[line 198]: “This is likely because with an increase in traversals, the Poisson noise of the non-place cells will increasingly average out over the traversals, thus resulting in an increased correlation between the first and second halves of the non-place cells’ fluorescence maps. This increases the number of false positives, and thus results in a decrease in specificity.”

[line 668]: “Furthermore, the Stability method increasingly falsely characterised non-place cells as place cells as the number of traversals increased, because non-place cells had higher intracell correlations as the number of traversals increased, and were thus wrongly classified as place cells”

5. It would be useful to have more quantitative data on the synthetic place cells used in Figs 1 and 2 beside the size of the place field for example of the amount of out versus in field firing, the reliability and spatial variability. It should ideally be comparable with place cells reported with electrophysiological recordings in rats and mice.

We thank the reviewer for this suggestion and think it is a very good idea. We have now added such quantitative data for the various place cell models across Figs 1, 2 and 3 in a new supplementary figure (S3 Fig). This figure shows the full width at half maximum (an approximation of place cell width), stability, mutual information and out/in ratio for modelled place cells with varying numbers of traversals, place field widths, peak size, variability and reliability.

6. The authors state that detection methods used with electrophysiological recordings cannot be adapted to data obtain with imaging techniques but it would be interesting for readers to determine how detection methods more classically used with electrophysiological recordings perform on the model dataset and how the population of detected place cells overlap with that of place cells detected with other methods. It would also widen the impact of the paper by extending its conclusions to data obtained with electrophysiological recordings.

We are sorry if this was unclear, as we believe we have already achieved this with our manuscript, by selecting the Peak and Stability methods. The Peak method was designed for use on electrophysiological data, and we adapted it for use on fluorescence data. The Stability method was based on methods used to characterize place cells in electrophysiological studies (e.g. [4,5]), which was adapted previously [6] to be used for fluorescence data. Most other approaches for analysing electrophysiological data use a threshold for the absolute firing rate (e.g. only cells with a peak firing rate above 1Hz are included [4,5], or use the shape of the waveform to include cells [5]). These measures cannot be extended to fluorescence data, because the exact spiking rates and waveforms cannot be extracted from the fluorescence data (we further expand upon this in our response to your next comment).

As this was unclear, we have now added additional clarification for the choice of these methods, as well as the choice of the Peak method as our preferred method, which hopefully clarifies the link to electrophysiological studies.

[line 135]: “We selected the four methods for varying reasons. The Peak method was originally applied to electrophysiological data, and its simplicity allows for the application to fluorescence data with minimal adaptations. Similarly, the Stability method was based on methods used for characterising place cells in electrophysiological recordings (e.g. [3]), while the Information method, or variations thereof, has previously been used to detect place cells in both electrophysiological [26] and fluorescence data [27]. This makes these three methods promising candidates for application across the two recording paradigms. In contrast, the Combination methods was designed specifically for fluorescence data, and has been widely employed in the field (e.g. [11,20,25]).”

[line 740]: “In addition, it has previously been successfully applied to electrophysiological recordings, and we therefore would predict that the properties of place cells in imaging data detected using the Peak method will most accurately reflect the properties of place cells detected in a similar manner in electrophysiological studies.”

7. Some imaging techniques use deconvolution to infer spikes trains from imaging data. It is unclear how good are spike train inferred from imaging and deconvolution methods but recent report suggests that they could be quite accurate. Then an important question is whether place cell detection would be better from the deconvoluted spike trains than from raw calcium data. Could the authors use their model dataset to provide answers to this question?

The accuracy of deconvolution is still contentious (e.g. [7,8]) so we would have to be very confident it would add to our results to consider it here, at a stage where its accuracy is still an active discussion in the community. Furthermore, we do not have a ground truth electrophysiology dataset, and our modelled datasets are likely distinct enough from true fluorescence data for meaningful deconvolution from these simulated signals to be infeasible. We do not therefore think that convolving the datasets to estimate spike firing would result in increased confidence in the performance of place cell detection. In addition, there is very little hypothetical gain from using deconvolution, as the same level of mutual information is found between place cells and location in fluorescence traces as in electrophysiological recordings [9]. In fact, adding an extra step in the processing provides the opportunity for more discrepancies between labs, for example, if they use different methods of deconvolution. Therefore, for simplicity, it is better to work as close to the data as possible, and apply the place cell detection directly on the fluorescence data. We believe our manuscript shows convincingly that these methods are indeed capable of classifying place cells satisfactorily.

8. Often a fixed velocity threshold is used to separate periods of movements (included in place cells analysis) from periods of immobility (excluded from place field analysis). Notably some of the methods analyzed here are performed using a fixed threshold. It is thus unclear why a variable threshold (10 % of maximum velocity) was used. Depending on the running behavior of the mice this will lead to highly different thresholds for movement detection (for example 4 cm/s in a fast running mice/session compared to 0.5 cm/s in a slow running mice/session). What is the impact of fixed versus variable velocity threshold on the results.

This is a very good point. We agree that a fixed threshold is the more appropriate way to include periods of running and should have used this from the outset. We have now altered the threshold to 2 cm/s for all of our analyses. This has caused changes to some of the results, most notably an increase in place cells detected by the Combination method in the real datasets, (Fig 5), as well as a small shift in their characteristics (Fig 6) but no changes to any of the conclusions.

Minor:

1. Fig. 1 could you illustrate trial by trial activity of non-place cells for comparison?

We have added a figure to illustrate the non-place cell activity to Fig 1, and a figure illustrating the activity of a non-place cell with random calcium peaks has been added to S7 Fig.

2. Fig. 2 could you show example of place fields with different width including 200 cm width.

We have added S Fig 3, with examples of place fields of varying widths.

References

1.	Mehta MR, Quirk MC, Wilson MA. Experience-dependent asymmetric shape of hippocampal receptive fields. Neuron. 2000;25(3):707–15.

2.	Gauthier JL, Tank DW. A dedicated population for reward coding in the hippocampus. Neuron. 2018;99(1):179–93.

3.	Dong C, Madar AD, Sheffield MEJ. Distinct place cell dynamics in CA1 and CA3 encode experience in new environments. Nat Commun. 2021;12(1):1–13.

4.	Anderson MI, Jeffery KJ. Heterogeneous modulation of place cell firing by changes in context. J Neurosci. 2003;23(26):8827–35.

5.	Grieves RM, Jedidi-Ayoub S, Mishchanchuk K, Liu A, Renaudineau S, Jeffery KJ. The place-cell representation of volumetric space in rats. Nat Commun. 2020;11(1):1–13.

6.	O’Leary A, Muessig L, Wills T, Cacucci F, Barry C. Cortical and Cortico-Hippocampal Circuits: Spatial Navigation III. Soc Neurosci 2019. 2019;Chicago, I(2019 Neuroscience Meeting Planner).

7.	Huang L, Ledochowitsch P, Knoblich U, Lecoq J, Murphy GJ, Reid C, et al. Relationship between simultaneously recorded spiking activity and fluorescence signal in GCaMP6 transgenic mice. Elife. 2021;10:e51675.

8.	Siegle JH, Ledochowitsch P, Jia X, Millman D, Ocker GK, Caldejon S, et al. Reconciling functional differences in populations of neurons recorded with two-photon imaging and electrophysiology. bioRxiv. 2020;

9.	Climer JR, Dombeck DA. Information theoretic approaches to deciphering the neural code with functional fluorescence imaging. bioRxiv. 2020;
